# Analysis of Sorafenib Outcome: Focusing on the Clinical Course in Patients with Hepatocellular Carcinoma

**DOI:** 10.1371/journal.pone.0161303

**Published:** 2016-08-18

**Authors:** Sadahisa Ogasawara, Tetsuhiro Chiba, Yoshihiko Ooka, Eiichiro Suzuki, Masanori Inoue, Toru Wakamatsu, Akinobu Tawada, Osamu Yokosuka

**Affiliations:** Department of Gastroenterology and Nephrology, Graduate School of Medicine, Chiba University, Chiba, Japan; National Yang-Ming University, TAIWAN

## Abstract

**Background:**

Treatment outcomes of sorafenib therapy may greatly vary depending not only on tumor spread but also on past clinical processes prior to sorafenib therapy and timing of sorafenib administration in the past clinical course of hepatocellular carcinoma (HCC). We evaluated the efficacy of sorafenib in patients with HCC, taking into account of their past clinical courses.

**Methods:**

Patients with HCC treated with sorafenib as a first-line systemic therapy, whose courses documented from the time of the initial diagnosis, were retrospectively analyzed.

**Results:**

Of the 123 patients receiving sorafenib therapy at an advanced-stage, baseline characteristics differed including the rate of hepatitis C virus, Child–Pugh class, and status of intrahepatic lesions according to stage progression processes. Overall survival (OS) in patients progressed directly from the early-stage (15.3 months) was significantly longer than that in patients diagnosed at the advanced-stage (5.3 months, P = 0.022) and progressed from the intermediate-stages (6.0 months, P = 0.041). Of 105 patients diagnosed at the intermediate-stage on past clinical courses, OS of starting sorafenib therapy before progression to the advanced-stage (67 patients) was significantly longer than for patients starting sorafenib therapy only after progression to the advanced-stage (38 patients) (P = 0.015).

**Conclusion:**

Characteristic differences between past stage progression processes might affect prognosis in advanced-stage HCC patients receiving sorafenib. Switching to sorafenib therapy before progression to the advanced-stage appears more effective than that after progression to the advanced-stage in patients diagnosed in the intermediate-stage on past clinical courses prior to sorafenib administration.

## Introduction

Hepatocellular carcinoma (HCC) is the third most common cause of cancer-related deaths and the 16th overall cause of deaths globally [1]. Approximately 90% of HCCs are associated with underlying cirrhosis mainly caused by hepatitis B virus (HBV) infection, hepatitis C virus (HCV) infection, alcohol consumption, and metabolic syndrome [2]. Hence, HCC mostly develops in damaged livers that exhibit a high potential of hepatocarcinogenesis.

According to data from the surveillance of high-risk populations, numerous patients are diagnosed with early-stage HCC and are eligible for potentially curative therapies, which include liver resection or local ablation [3–6]. Nowadays, curative therapies extend survival by more than 60 months [7, 8]. Unfortunately, the majority of patients have developed recurrence within 5 years because of a high risk of hepatocarcinogenesis and spread from the primary tumors [7–9]. Thus, quite a few patients progress into the intermediate-stage or directly into the advanced-stage from the early-stage. Transarterial chemoembolization (TACE) is a widely recommended treatment strategy for the intermediate-stage [3–6]. Treatment with TACE for intermediate-stage HCC is associated with an estimated median survival of 26.1–27.8 months [10, 11]. However, TACE is often repeated, and only few patients are completely cured. Repeated TACE promotes resistance and increases the chance of tumor recurrence and progression to the advanced-stage.

Presently, sorafenib is recommended as a first-line systemic therapy treatment option in patients with advanced-stage HCC based on the results of two-phase III studies [12, 13]. Moreover, several guidelines have advocated that patients with intermediate-stage HCC are eligible for sorafenib treatment in the case of TACE treatment failure [4, 14, 15]. Consequently, patients who are initially diagnosed with advanced-stage HCC, progressing from the early- or intermediate-stage to the advanced-stage, and intermediate-stage patients who are deemed refractory to TACE are offered sorafenib therapy in field practice. Although applicable patients of sorafenib therapy are broad and the clinical processes before starting sorafenib are various, treatment outcomes of sorafenib can vary widely. This variability may depend not only on the manner of tumor spread but also on the clinical processes implemented prior to sorafenib therapy and timing of sorafenib administration in the clinical course of HCC. Additionally, whether to convert to sorafenib therapy from TACE before or after progression to the advanced-stage in intermediate-stage HCC patients is still controversial. However, little has been reported on the effectiveness of sorafenib therapy while taking the clinical course of HCC into account.

The goals of this study were as follows: (1) to characterize and assess sorafenib-treated patients focusing on stage progressing processes from initial diagnosis prior to sorafenib therapy and (2) to compare data of patients diagnosed in intermediate-stage HCC on past clinical course prior to sorafenib therapy who are switched from TACE to sorafenib therapy before and after progressing to advanced-stage HCC, respectively.

## Patients and Methods

### Ethics statement

This study was approved by the Research Ethics Committees of Graduate School of Medicine, Chiba University (approval number 2,250). Informed consent was not obtained because of the retrospective design. Patient records/information were anonymized and de-identified prior to analysis.

### Patients

A total of 229 patients with HCC were consecutively treated with sorafenib as first-line systemic therapy between June 2009 and December 2014 at Chiba University Hospital, Japan. Among these, 39 patients were excluded due to one of the following reasons: (1) lack of data on the clinical process from the time of initial diagnosis (n = 27); (2) no TACE therapy after being diagnosed with intermediate-stage HCC, which is the standard therapy for intermediate-stage HCC (n = 12). Eventually, this study included 190 patients who received sorafenib as first-line systemic therapy and for whom data on the clinical process from the time of initial diagnosis were available, including liver function, tumor marker (alfa-fetoprotein [AFP]), and radiological assessment. For all patients, the presence of histologically confirmed or clinically diagnosed HCC could be documented (fulfilling the criteria for lesions with typical imaging) [6].

### Sorafenib treatment

The treatment policy for patients with HCC followed the consensus-based clinical practice guideline proposed by the Japan Society of Hepatology (JSH) [4]. The timing of conversion from TACE to sorafenib was generally judged in the specialist’s meeting based on the JSH guideline [4].

A total of 400 mg of sorafenib was orally administered twice per day (full dose = 800 mg). However, the initial dose of sorafenib was subject to reduction at the treating physician’s discretion based on liver function and/or age. Sorafenib dose reductions (400 mg once daily or 400 mg on alternate days) and interruptions were allowed and depended on the type and severity of adverse events. Decisions on dose reduction and treatment discontinuation were informed by package insert data and experience with drug-related toxicities. We continued sorafenib administration until the development of intolerable toxicity or clear clinical disease progression. In our institution, the standard radiological follow-up procedure in sorafenib-treated patients was baseline, followed by evaluation in the first month after treatment, and then every 2 months. Because there is no satisfactory evidence in combination therapy, we did not combine sorafenib and other treatment such as TACE. If patients showed disease progression after sorafenib therapy, we considered whether definitive clinical trial could be accomplished, initially. All remaining cases are considered for best supportive care or sub-optimal treatments which were approved by the Ministry of Health, Labour and Welfare and were covered by social insurance of Japan, although there was no evidence-based treatment after sorafenib failure.

### Clinical parameters

Clinical parameters included: baseline demographic data [gender, age, etiology, Eastern Cooperative Oncology Group performance status (ECOG-PS), Child–Pugh class, radiological assessment, AFP, treatment prior to the initiation of sorafenib therapy, and initial dose], adverse events after starting sorafenib, average daily dose, date of radiological progression according to the Response Evaluation Criteria in Solid Tumors (RECIST) version 1.1 [16], progression pattern, liver function deterioration at the end of sorafenib treatment, post-sorafenib therapy, and date of death or last follow-up. Data were collected via the database of sorafenib-treated patients in our institution. We also reviewed medical records to identify the history of the clinical course of HCC prior to sorafenib administration and the initial diagnosis of each patient (i.e., early-, intermediate- or advanced-stage HCC). We then adjusted the clinical parameters at the time of diagnosis at the intermediate-stage (age, Child–Pugh class, radiological assessment, and AFP) and effectiveness of initial TACE in the intermediate-stage according to the modified RECIST (mRECIST) [17]. Patients responding to initial TACE were defined as patients who had achieved a partial or a complete response for more than 3 months after TACE. Data on ECOG-PS for patients diagnosed with intermediate-stage were removed from the final data set available for analysis because they could not be identified objectively from the medical charts.

In this study, we defined each HCC stage as follows: (1) early-stage: single lesion of any size or 2–3 lesions of ≤30 mm diameter; (2) intermediate-stage: >3 lesions of any size or 2–3 lesions of >30 mm diameter; (3) advanced-stage: macrovascular invasion (MVI) or extrahepatic metastasis (EHM). Stage progression processes of patients starting sorafenib therapy at the advanced-stage were classified into three categories (advanced-stage at the time of initial diagnosis, progression to advanced-stage through the intermediate-stage, progression to the advanced-stage directly from the early-stage). Intermediate-stage patients were also classified into two groups as follows: those who had already developed intermediate-stage HCC upon initial diagnosis and those who had progressed from the early-stage.

### Statistical analysis

The chi-square test or Fisher’s exact test was used to compare demographic and clinical characteristics as appropriate. Kaplan–Meier plots of medians [with 95% confidence intervals (95% CI)] were used to estimate overall survival (OS). The censoring date was defined as the date of the last follow-up. Time to progression after sorafenib administration was estimated using Kaplan–Meier plots of medians (with 95% CI), with the censoring date being defined as the date of the last radiological assessment without progression. Univariate and multivariate Cox proportional-hazard models were used to estimate the hazard ratios for risk factors predicting OS. A probability (*P*) value of <0.05 was considered statistically significant. We defined a cut-off value of maximum size (≤50 mm/>50 mm) and number (≤7/>7) of intrahepatic lesions based on our previous study [11]. Propensity scores were used to generate matched pairs for comparisons of survival between the timing of sorafenib administration before and after progression to the advanced stage in patients with intermediate-stage HCC. Possible variables associated with survival of the patients with intermediate stage HCC, including tumor number, maximum size of the tumor, Child-Pugh class, AFP, and response to initial TACE, were comprehensively selected for propensity score generation. Logistic regression analysis with the selected variables was used to generate a continuous propensity score from 0 to 1. A one-to-one nearest neighbourhood match between the two groups was used to select patients for subsequent analysis. All statistical analyses were performed using SPSS statistical software (version 23; SPSS-IBM, Chicago, IL, USA).

## Results

### Patient characteristics at baseline of sorafenib administration

Table 1 displays the baseline characteristics of 190 enrolled patients with HCC. Most patients were males (77%), and the median age was 72 years (range, 35–85). The most frequent etiology was HCV (50%), followed by HBV (15%), and alcohol abuse (9%). One hundred thirty-eight patients (73%) were classified as Child–Pugh A, and 52 patients (27%) were classified as Child–Pugh B. Sixty-seven patients (35%) had MVI and 85 patients (45%) had EHM.

**Table 1:** Baseline demographic data and patient characteristics when starting sorafenib.

Demographics/characteristics	
**Gender** [*n* (%)]	
Male	146 (77)
Female	44 (23)
**Age, years** [*n* (%)]	
≤72	103 (54)
>72	87 (46)
Median (range)	72 (35–83)
**HBV** [*n* (%)]	
Absent	162 (85)
Present	28 (15)
**HCV** [*n* (%)]	
Absent	95 (50)
Present	95 (50)
**Alcohol abuse** [*n* (%)]	
Absent	173 (91)
Present	17 (9)
**ECOG-PS** [*n* (%)]	
0	96 (51)
≥1	94 (49)
**Child–Pugh score** [*n* (%)]	
A	138 (73)
B	52 (27)
**Intrahepatic lesions** [*n* (%)]	
Absent	13 (7)
Present	177 (93)
**Maximum size of the intrahepatic lesion, >50 mm** [*n* (%)]	
Absent	124 (65)
Present	66 (35)
**Number of intrahepatic lesions, >7** [*n* (%)]	
Absent	77 (41)
Present	113 (59)
**MVI** [*n* (%)]	
Absent	123 (65)
Present	67 (35)
**EHM** [*n* (%)]	
Absent	105 (55)
Present	85 (45)
**MVI or/and EHM** [*n* (%)]	
Absent	67 (35)
Present	123 (65)
**BCLC stage** [*n* (%)]	
B	41 (22)
C	149 (78)
**AFP, ng/mL** [*n* (%)]	
≤400	106 (56)
>400	84 (44)
**Pre-treatment** [*n* (%)]	
Absent	21 (11)
Present	169 (89)
**Initial dose of sorafenib, 800 mg/day**	
Absent	13 (7)
Present	177 (93)
**Average daily dose** [*n* (%)]	
>400 mg	98 (52)
≤400 mg	92 (48)

Abbreviations: HBV, hepatitis B virus; HCV, hepatitis C virus; ECOG-PS, Eastern Cooperative Oncology Group performance status; MVI, macrovascular invasion; EHM, extrahepatic metastasis; BCLC, Barcelona clinic liver cancer; AFP, alpha-fetoprotein

### Sorafenib therapy

Most of the patients (93%) received a full dose of sorafenib (800 mg/day). One hundred fifty-nine patients (84%) required dose modifications, and 29 patients (15%) and 5 patients (3%) discontinued sorafenib therapy because of severe adverse events and patient withdrawal, respectively. At the time of data collection (end of October 2015), 148 patients had died, 13 patients were still alive, and 29 patients were lost to follow-up. The median OS was 9.5 months (95% CI: 7.2–11.9), and the median TTP was 2.8 months (95% CI: 2.6–3.0).

### Stage progression process before sorafenib started

**Fig 1:**
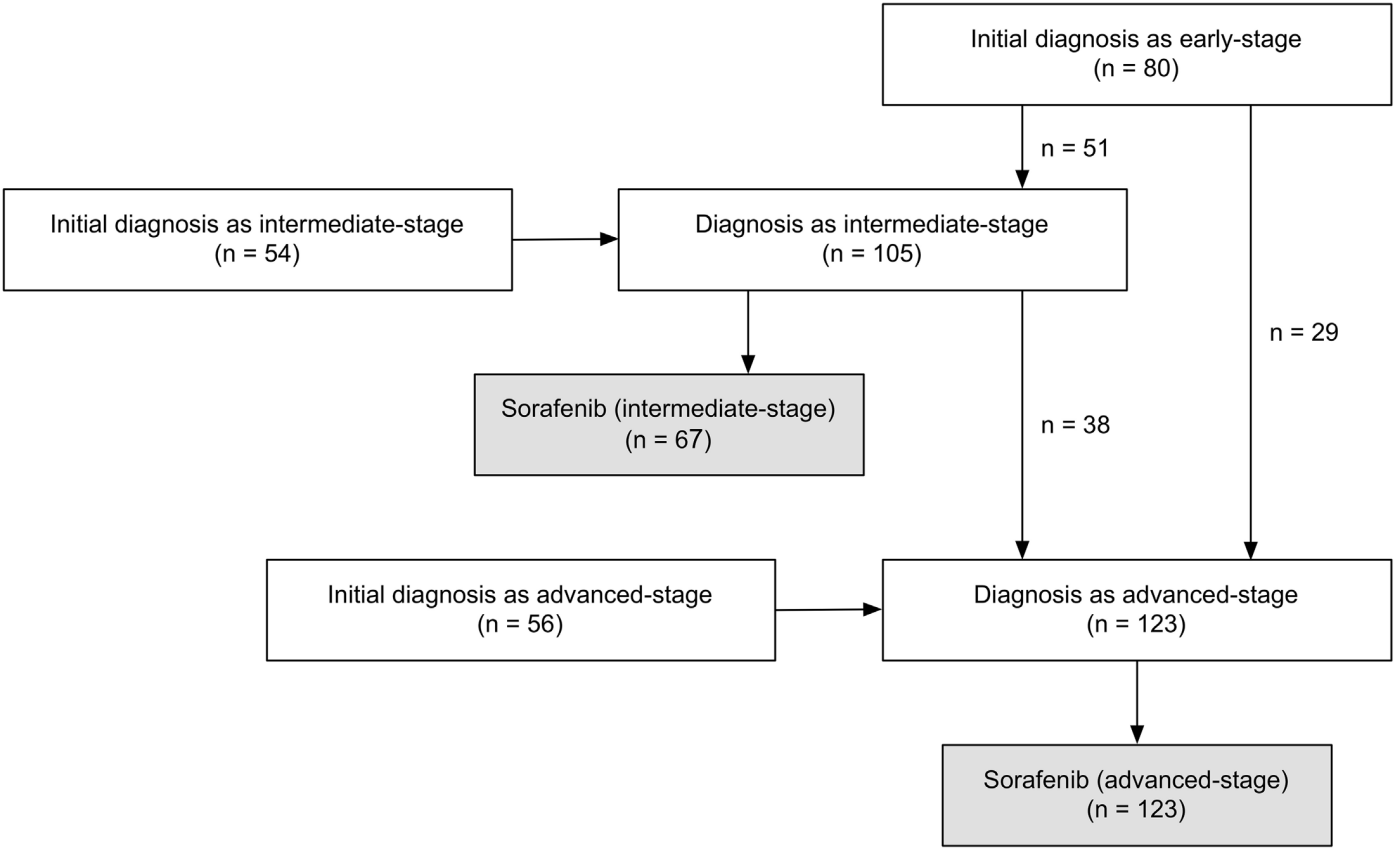
Initial diagnosis and stage progression processes of study patients.

Fig 1 demonstrates data pertaining to initial diagnosis and stage progression processes of the patients. The time from initial diagnosis to sorafenib administration was 14.3 months (95% CI: 9.8–18.8). The time from initial diagnosis to sorafenib administration in patients initially diagnosed with early-, intermediate-, and advanced-stage HCC was 48.7 (95% CI: 39.8–57.6), 10.3 (95% CI: 6.4–14.2), and 1.3 months (95% CI: 0.9–1.6), respectively.

### Sorafenib therapy in advanced-stage HCC patients

The baseline characteristics of patients starting on sorafenib therapy at advanced-stage HCC are included in S1 Table. Patients directly progressing from early-stage HCC had the highest rate of HCV infection (69%) compared with the proportion of patients diagnosed with the advanced-stage (25%) and those who progressed through the intermediate-stage (45%) (*P* < 0.001). The majority (75%) of patients initially diagnosed with advanced-stage HCC had intrahepatic MVI. Meanwhile, 31% of patients that directly progressed from the early-stage did not have intrahepatic lesions. Hence, the status of intrahepatic lesions significantly differed among the three groups (*P* < 0.001).

**Fig 2:**
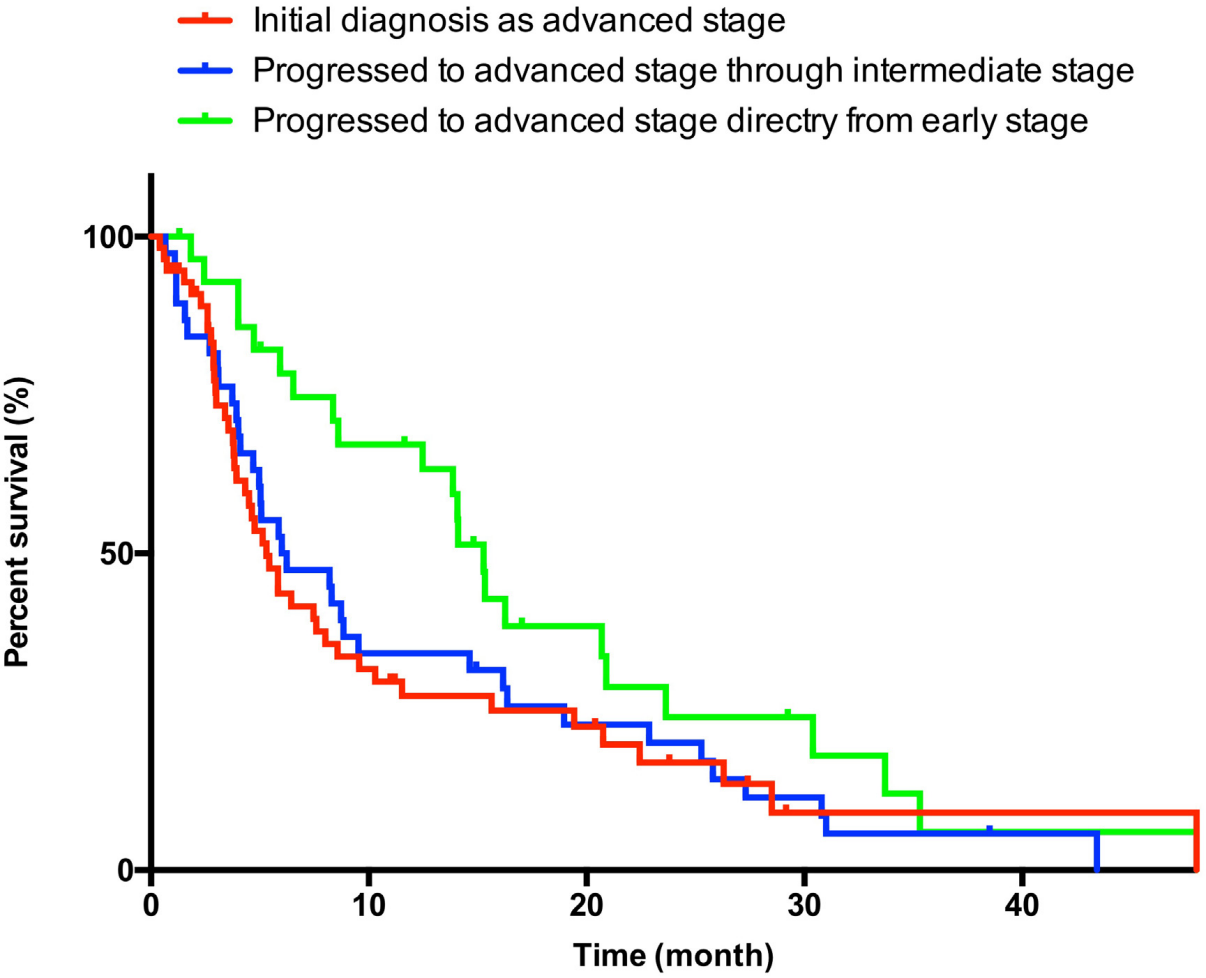
Kaplan–Meier survival curve in advanced-stage hepatocellular carcinoma patients receiving sorafenib.

The median OS in advanced-stage HCC patients was 7.6 months (95% CI: 5.4–9.8). Kaplan–Meier estimates of OS according to stage progression process are shown in Fig 2. The OS of patients initially diagnosed with advanced-stage HCC, progressing through the intermediate-stage, and directly progressing from the early-stage was 5.3 (95% CI: 3.9–6.7), 6.0 (95% CI: 1.3–10.7), and 15.3 months (95% CI: 12.2–18.5), respectively. Although there was no significant difference between patients initially diagnosed with advanced-stage and those who progressed through the intermediate-stage (*P* = 0.822), patients directly progressing from the early-stage HCC experienced significantly longer survival compared with the other two groups (vs. patients initially diagnosed with advanced-stage HCC: *P* = 0.022; vs. patients progressing through the intermediate-stage: *P* = 0.041). Multivariate analysis identified baseline age, ECOG-PS, status of intrahepatic lesions (none, without MVI, and with MVI), number of intrahepatic lesions as independent predictors of OS in patients with advanced-stage HCC (Table 2; univariate analysis are shown in S2 Table). Meanwhile, stage progression process had no predictive power in this analysis.

**Table 2:** Multivariate analysis of survival in sorafenib-treated patients with advanced-stage hepatocellular carcinoma.

Variables	Multivariate analysis		*P*
	Hazard ratio	95% CI	
**Age, >72 years**			
Absent	Reference		
Present	0.478	0.295–0.777	0.003
**ECOG-PS, >0**			
Absent	Reference		
Present	3.000	1.814–4.984	<0.001
**Status of intrahepatic lesions**			
None	1.214	0.529–2.784	0.647
Without MVI	Reference		
With MVI	2.616	1.644–4.162	<0.001
**Number of intrahepatic lesions, >7**			
Absent	Reference		
Present	2.300	1.484–3.565	<0.001

Abbreviations: ECOG-PS, Eastern Cooperative Oncology Group performance status; MVI, macrovascular invasion.

### Sorafenib therapy in intermediate-stage HCC patients

**Fig 3:**
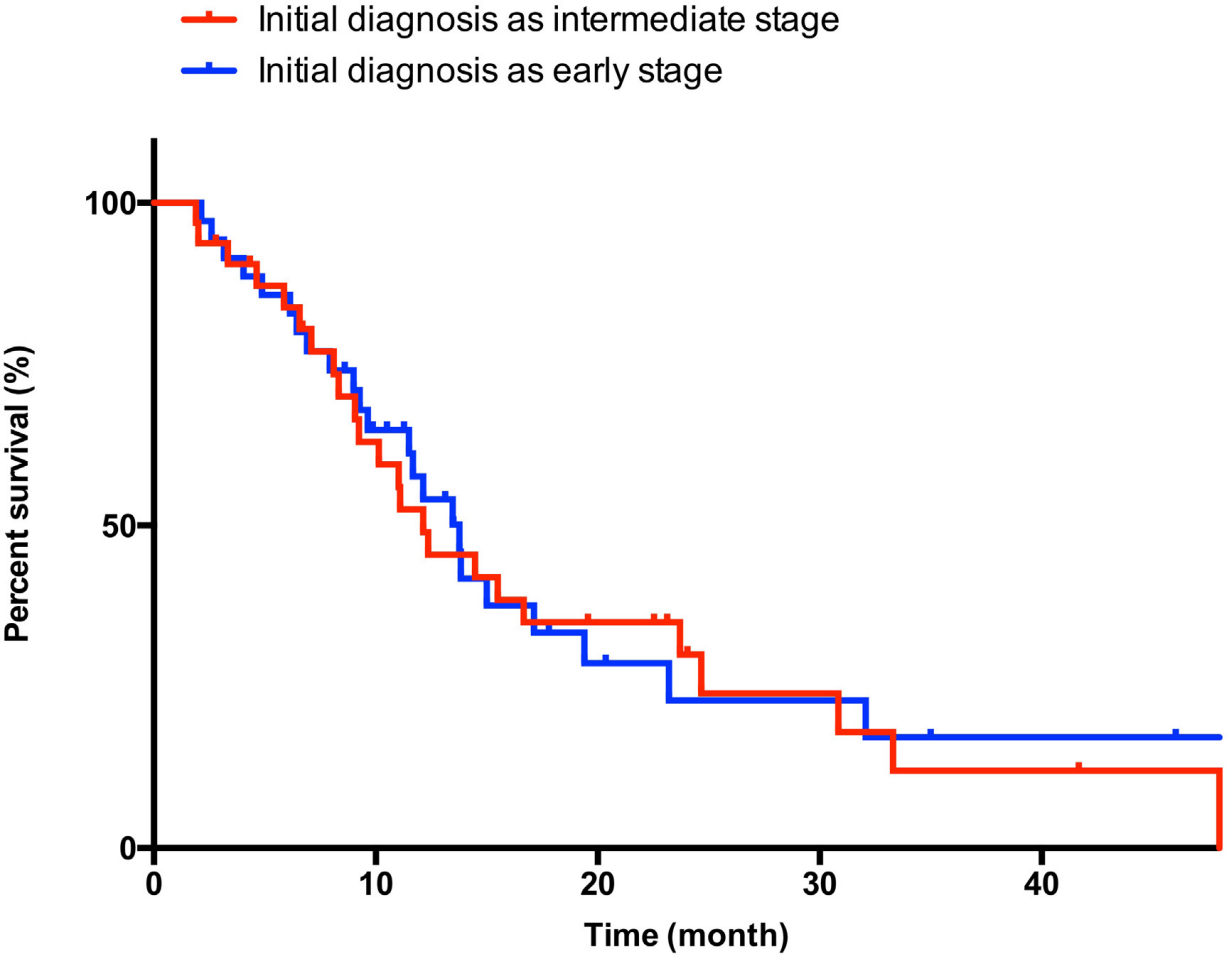
Kaplan–Meier survival curve in intermediate-stage hepatocellular carcinoma patients receiving sorafenib.

The baseline characteristics of patients starting on sorafenib at the intermediate-stage are included in S3 Table. Patients diagnosed with early-stage disease had a higher rate of HCV infection (83%) compared with the rate of patients diagnosed with intermediate-stage HCC (47%) (*P* = 0.004). No significant differences were observed for any of the other variables. In intermediate-stage HCC patients, OS upon sorafenib administration was similar among the different stages of the disease [Fig 3; any: 12.6 months (95% CI: 9.9–14.8); diagnosed with intermediate-stage HCC: 12.1 months (95% CI: 7.6–16.6); diagnosed with early-stage HCC: 13.8 months (95% CI: 11.3–16.2); *P* = 0.884]. Multivariate analysis identified baseline AFP as the only independent predictor of OS in patients with intermediate-stage HCC (Table 3; univariate analysis are shown in S4 Table).

**Table 3:** Multivariate analysis of survival in sorafenib-treated patients with intermediate-stage hepatocellular carcinoma.

Variables	Multivariate analysis		*P*
	Hazard ratio	95% CI	
**AFP, >400 ng/mL**			
Absent	Reference		
Present	3.474	1.839–6.562	<0.001

Abbreviations: AFP, alpha-fetoprotein

### Progression pattern of sorafenib treatment, liver function deterioration at the end of sorafenib treatment, and post-sorafenib therapy

S5 Table shows correlations of the OS and progression pattern of sorafenib according to previous reports [18–20], with liver function deterioration at the end of sorafenib treatment defined as either jaundice (total bilirubin >3.0 mg/dL), massive ascites, or encephalopathy. In advanced-stage HCC, patients who had new extrahepatic lesions and liver deterioration at the end of sorafenib treatment had significantly poor prognosis. On the other hand, in intermediate-stage HCC, patients who had new intrahepatic lesions and liver deterioration at the end of sorafenib treatment showed significantly poor prognosis. Moreover, majority of patients who had liver deterioration at the end of sorafenib treatment received best supportive care as post-sorafenib therapy [advanced-stage HCC: 45 of 48 patients (94%), intermediate-stage HCC: 11 of 13 patients (85%)].

### Impact of the timing of sorafenib administration before or after progression to the advanced stage in intermediate-stage HCC patients

**Fig 4:**
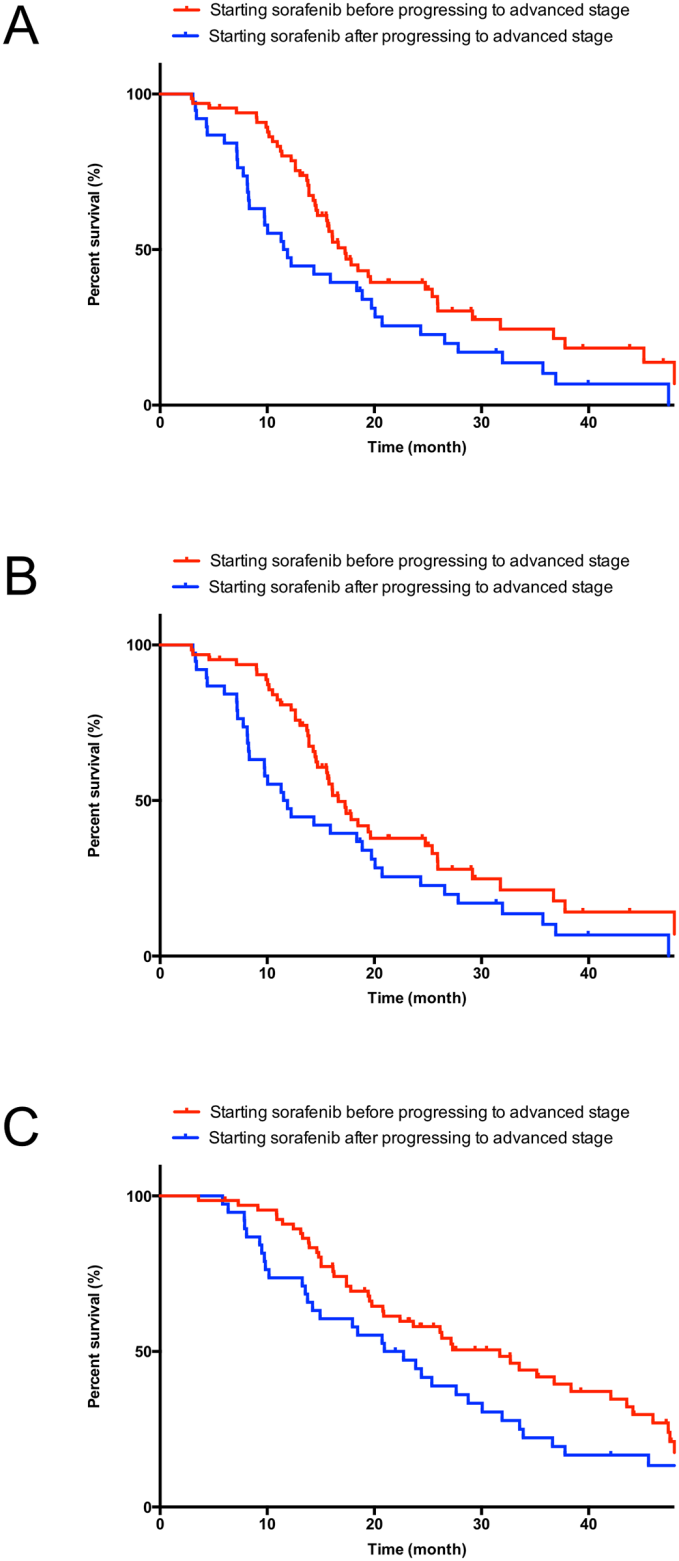
Kaplan-Meier survival curve in patients diagnosed with intermediate-stage hepatocellular carcinoma. A: the day from the last TACE (any patient), B: the day from the last TACE (patients deemed as TACE refractory/failure), and C: the day from the point of being diagnosed with intermediate-stage HCC.

Demographic data and other characteristics for the 105 patients who were diagnosed with intermediate-stage disease prior to commencing sorafenib therapy are included in Table 4. All 105 patients received TACE at least once after the diagnosis of intermediate-stage HCC [median 2, (range, 1–10)]. Upon analysis of demographic data and other characteristics for these patients, no significant difference was observed between starting sorafenib before or after progressing to advanced-stage HCC, including sub-classification model of intermediate-stage [21]. Of 67 patients who started sorafenib before progressing to advanced-stage HCC, 64 patients (96%) were deemed as TACE failure/refractory according to the JSH guideline before conversion from TACE to sorafenib [4]. Of 38 patients who started sorafenib after progressing to advanced-stage HCC, 24 patients (63%) deemed as TACE failure/refractory without MVI and EHM. These 24 patients continued TACE even if they deemed TACE failure/refractory and started sorafenib after progressing to advanced-stage HCC. The remaining 14 patients (37%) were deemed TACE failure/refractory with either or both of MVI and EHM. The median time from the day of last TACE to advanced-stage HCC was 3.2 months (95%CI: 2.8–3.6). The median survival from the day of the last TACE was significantly longer than in patients who started sorafenib before progressing to advanced-stage HCC than in those who started sorafenib after progressing to advanced-stage HCC [Fig 4A (any patient), starting sorafenib before progressing to advanced-stage HCC: 17.3 months (95% CI: 14.6–19.9) and starting sorafenib after progressing to advanced-stage HCC: 11.5 months (95% CI: 8.2–14.9); *P* = 0.022 and Fig 4B (patients deemed as TACE failure/refractory), starting sorafenib before progressing to advanced-stage HCC: 16.6 months (95% CI: 14.3–19.0) and starting sorafenib after progressing to advanced-stage HCC: 11.5 months (95% CI: 8.2–14.9); *P* = 0.040]. The OS from the point of being diagnosed with intermediate-stage HCC in patients starting sorafenib before progressing to advanced-stage disease was significantly longer than in those starting sorafenib after progressing to advanced-stage HCC [Fig 4C, starting sorafenib before progressing to advanced-stage HCC: 31.7 months (95% CI: 23.9–39.4); starting sorafenib after progressing to advanced-stage HCC: 20.9 months (95% CI: 12.9–28.9); *P* = 0.015]. TTP of sorafenib treatment in patients starting sorafenib before and after progressing to advanced-stage HCC was 3.5 months (95% CI: 2.7–4.2) and 1.6 months (95% CI: 0.6–2.7), respectively (*P* = 0.130). In patients starting sorafenib before progressing to advanced stage, 11 patients (16%) and 20 patients (30%) progressed to advanced-stage at the time of defined as disease progression and until the last radiological assessment of follow up period [median time from the point of being diagnosed with intermediate-stage to advanced-stage HCC: not reached, median follow up from the point of being diagnosed with intermediate-stage HCC: 20.5 months (14.6–26.5)]. Although selection bias between two groups might exist, we also created propensity score matched dataset (S6 Table). Multivariate analysis of both any patient and propensity matched dataset identified the timing of sorafenib administration as an independent predictor of OS in patients with intermediate-stage HCC, as well as the effectiveness of initial TACE in intermediate-stage HCC (Table 5; univariate analysis are shown in S7 Table).

**Table 4:** Baseline demographic data and patient characteristics at the time of being diagnosed with intermediate-stage hepatocellular carcinoma.

	All patients	Starting sorafenib before progressing to advanced stage	Starting sorafenib after progressing to advanced stage	*P*
**Number of patients**	105	67	38	
**Gender** [*n* (%)]				
Male	81 (77)	49 (73)	32 (84)	0.233
Female	24 (23)	18 (27)	6 (16)	
**Age, years** [*n* (%)]				
≤72	64 (61)	41 (61)	23 (61)	1.000
>72	41 (39)	26 (39)	15 (39)	
**HBV** [*n* (%)]				
Absent	94 (90)	62 (93)	32 (84)	0.200
Present	11 (10)	5 (7)	6 (16)	
**HCV** [*n* (%)]				
Absent	44 (42)	23 (34)	21 (55)	0.481
Present	61 (58)	44 (66)	17 (45)	
**Alcohol abuse** [*n* (%)]				
Absent	98 (93)	65 (97)	33 (87)	1.000
Present	7 (7)	2 (3)	5 (13)	
**Child–Pugh** [*n* (%)]				
A	96 (91)	61 (91)	35 (92)	1.000
B	9 (9)	6 (9)	3 (8)	
**Tumor size, >50 mm**[*n* (%)]				
Absent	77 (73)	49 (73)	28 (74)	1.000
Present	28 (27)	18 (27)	10 (26)	
**Tumor Number, >7** [*n* (%)]				
Absent	65 (62)	38 (57)	27 (71)	0.209
Present	40 (38)	29 (43)	11 (29)	
**Sub-classification of BCLC B** [*n* (%)]				
B1	33 (31)	22 (33)	11 (29)	0.509
B2	66 (63)	42 (63)	24 (63)	
B3	5 (5)	3 (4)	2 (5)	
B4	1 (1)	0 (0)	1 (3)	
**AFP, ng/mL** [*n* (%)]				
≤400	74 (70)	49 (73)	25 (66)	0.506
>400	31 (30)	18 (27)	13 (34)	
**Initial diagnosis** [*n* (%)]				
Early stage	50 (48)	35 (52)	15 (39)	0.229
Intermediate stage	55 (52)	32 (48)	23 (61)	
**Initial dose of sorafenib, 800 mg/day** [*n* (%)]				
Absent	7 (7)	5 (7)	2 (5)	1.000
Present	98 (93)	62 (93)	36 (95)	
**Effectiveness of initial TACE in intermediate stage**[*n* (%)]				
Responder	64 (61)	40 (60)	24 (63)	0836
Non-responder	41 (39)	27 (40)	14 (37)	

Abbreviations: HBV, hepatitis B virus; HCV, hepatitis C virus; BCLC, Barcelona clinic liver cancer; AFP, alpha-fetoprotein; TACE, transcatheter arterial chemoembolization

**Table 5:** Multivariate analysis of survival after being diagnosed with intermediate-stage hepatocellular carcinoma.

Variables	Multivariate analysis		*P*
	Hazard ratio	95% CI	
**Any patients (n = 105)**			
**Effectiveness of initial TACE in intermediate stage**			
Responder	Reference		
Non-responder	3.080	1.920–4.942	<0.001
**Administration timing of sorafenib**			
Conversion before progressing to advanced stage	Reference		
Conversion after progressing to advanced stage	1.808	1.156–2.829	0.009
**Propensity score matched dataset (n = 72)**			
**Effectiveness of initial TACE in intermediate stage**			
Responder	Reference		
Non-responder	2.754	1.570–4.829	< 0.001
**Administration timing of sorafenib**			
Conversion before progressing to advanced stage	Reference		
Conversion after progressing to advanced stage	1.905	1.133–3.203	0.015

Abbreviations: HBV, hepatitis B virus; HCV, hepatitis C virus; AFP, alpha-fetoprotein; TACE, transarterial chemoembolization.

## Discussion

Focusing on the clinical course of HCC until the administration of sorafenib, we attempted to organize the heterogeneous population of sorafenib-treated patients. This approach differed from previous studies that have evaluated the outcome of sorafenib-treated HCC patients in field practice [22–26] and might give us a new perspective.

Our study showed that the baseline characteristics in sorafenib-treated patients with advanced-stage HCC differed among patient groups with different stage progression processes. Patients initially diagnosed with advanced-stage HCC had significantly high rates of i) Child-Pugh B class, ii) intrahepatic lesions with MVI, and iii) absence of HCV infection. The majority of these patients, particularly patients in whom HCC is not associated with virus infection, might not have been subjected to HCC screening. Thus, they were diagnosed with advanced-stage HCC with highly progressing and decreasing liver function. On the other hand, we have often experienced HCC patients who recur with EHM but with or without only minor intrahepatic lesions upon curative therapy. Of importance, these patients were not complicated by MVI in many cases. Our results confirmed that 62% of patients directly progressing from the early-stage disease either did not have intrahepatic lesions (31%) or had intrahepatic lesions without MVI (31%). Progression through the intermediate-stage to the advanced-stage is a common clinical course in HCC. This study showed that intrahepatic lesions were more frequently accompanied by MVI than that of the other two groups. Moreover, the rate of HCV and Child–Pugh B class were between the other two categories. Although all of them were classified as having advanced-stage HCC treated with sorafenib, they were regarded as clinically different categories. Based on a meta-analysis of four randomized control trial, Shao YY reported that sorafenib might provide survival benefits to patients positive for HCV [27]. However, our results might indicate that differences in the rate of HCV between stage progression processes influenced that result.

Next, we performed separate analyses of the prognosis of sorafenib therapy in intermediate- and advanced-stage patients. The results of the multivariate analysis of patients with advanced-stage HCC demonstrated that the status of intrahepatic lesions was an independent prognostic factor. Several studies have indicated that the status of intrahepatic lesions and/or MVI were significant predictors of survival in patients with EHM [28, 29]. Sohn et al. reported that patients characterized by intrahepatic lesions with MVI experienced shorter survival times compared with patients characterized by intrahepatic lesions without MVI, and patients harboring intrahepatic lesions in sorafenib treated patients with EHM [23]. Considering these findings, status of intrahepatic lesions, particularly the presence of MVI, were strong, poor predictive factors for sorafenib-treated patients with advanced-stage HCC. Stage progression process from initial diagnosis appeared to be associated with the status of intrahepatic lesions in advanced-stage HCC. Our results indicated that the OS of patients directly progressing from early-stage HCC was significantly longer than that observed for the other two categories. Although stage progression process was not an independent prognosis factor, we believe that the differences in clinical characteristics among the three categories, particularly the status of intrahepatic lesions, might affect patient prognosis.

In intermediate-stage patients who started sorafenib, our results identified AFP as the only independent prognostic factor. Previous studies have shown that the maximum size and/or number of intrahepatic lesions are predictive factors of intermediate-stage HCC patients who have received TACE [11, 30, 31]. Thus, the prognosis of intermediate-stage patients is thought to depend on these two factors as well as liver function [21, 32]. In contrast, our finding that both maximum tumor size and number were not predictive factors of survival in sorafenib-treated patients. This might be relevant for considering treatment strategies for intermediate-stage HCC patients. Prior to this study, there have only been a few studies that evaluated the outcome of sorafenib therapy, specifically with regard to intermediate-stage patients [18, 33], and this is the first report to analyze prognostic factors of sorafenib therapy in intermediate-stage HCC patients. In this analysis, ECOG-PS and Child-Pugh class were not independent prognostic factors in spite of being significant factors of the univariate analysis. These factors have been known to contribute to the survival after sorafenib therapy [22, 34]. Although our result was from a small number and a single institution, additional analyses should be conducted to confirm the outcome of sorafenib treatment in patients with intermediate-stage HCC.

This study identified that switching to sorafenib before progression to advanced-stage disease conferred a survival benefit compared with switching to sorafenib only after progression to advanced-stage disease in patients diagnosed in intermediate-stage HCC on the past clinical course prior to sorafenib therapy. Although this analysis was based on retrospective data and it might have selection bias and lead time bias of conversion from TACE to sorafenib, our results indicate that the difference in timing of switching to sorafenib was associated with the outcome of the two groups. After the approval of sorafenib, several suggestions for alternative therapies in patients with TACE failure/refractoriness have been made [4, 14, 15]. Moreover, two reports demonstrated that switching to sorafenib upon TACE failure according to the definition of TACE failure/refractoriness conferred longer survival compared with continued TACE in patients with intermediate-stage HCC [35, 36]. These findings, coupled with our results, could indicate that switching to sorafenib from TACE in intermediate-stage HCC patients is an effective treatment strategy for patients diagnosed with intermediate-stage HCC compared with continuing TACE until progression to advanced-stage HCC.

For major solid cancers, decisions on treatment often rely simply on whether they are “resectable” or “unresectable,” and first-line systemic therapies are initial treatments for “unresectable” patients (NCCN Gudelines^®^; http://www.nccn.org). With HCC, the heterogeneous clinical courses and treatment processes associated with the disease complicate the picture as follows compared with major solid cancers. First, HCC has a high potential of recurrence because of primary tumor spread and persisting hepatocarcinogenesis [9]. Second, there are several treatment options for “unresectable” patients such as local ablation, TACE, and sorafenib therapy [4–6]. Patients typically receive treatments that are considered suitable at that particular time of HCC progression. Finally, liver function is a strong prognostic factor and needs to be considered before making treatment choices [4–6]. Because it is not feasible to use sorafenib as an initial treatment option in most patients [22, 23, 25, 34], the clinical course before starting sorafenib and analyzing the outcome of sorafenib therapy in heterogeneous populations should be considered.

In conclusion, the characteristics of patients receiving sorafenib were different according to stage progression process from the time of initial diagnosis. These factors might affect the prognosis of sorafenib therapy in advanced-stage HCC patients receiving sorafenib. Switching to sorafenib from TACE before progression to advanced-stage HCC appears to be effective compared with switching to sorafenib only after progression to the advanced-stage. Additional, large-scale studies that take into account the clinical course of HCC are needed to identify the suitable timing of sorafenib administration over the clinical course of HCC.

## Supporting Information

S1 Table Baseline demographic data of patient characteristics at the time of starting sorafenib (advanced-stage hepatocellular carcinoma patients). (PDF)

S2 Table Univariate analysis of survival in sorafenib-treated patients with advanced-stage hepatocellular carcinoma. (PDF)

S3 Table Baseline demographic data and patient characteristics at the time of starting sorafenib (intermediate-stage hepatocellular carcinoma patients). (PDF)

S4 Table Univariate analysis of survival in sorafenib-treated patients with intermediate-stage hepatocellular carcinoma. (PDF)

S5 Table Progression pattern of sorafenib treatment, liver function deterioration at the end of sorafenib treatment, and post-sorafenib therapy. (PDF)

S6 Table Baseline demographic data and patient characteristics at the time of being diagnosed with intermediate-stage hepatocellular carcinoma (propensity score matched dataset). (PDF)

S7 Table Univariate analysis of survival after being diagnosed with intermediate stage hepatocellular carcinoma. (PDF)

S8 Table Clinical data of the study participants (baseline). (XLSX)

S9 Table Clinical data of the study participants (being diagnosed with intermediate stage hepatocellular carcinoma). (XLSX)

S10 Table Clinical data of the study participants (being diagnosed with intermediate stage hepatocellular carcinoma; propensity score matched dataset). (XLSX)
