# Supplementary material for: Analysis of Sorafenib Outcome: Focusing on the Clinical Course in Patients with Hepatocellular Carcinoma
Source: PLoS One. 2016 Aug 18;11(8):e0161303. doi: 10.1371/journal.pone.0161303 (PMC4990272; doi:10.1371/journal.pone.0161303)
Supplement: S1 Table — (PDF) [file pone.0161303.s001.pdf]

S1 Table. Baseline demographic data and patient characteristics at the time of starting sorafenib (advanced-stage hepatocellular carcinoma patients)

	All patients	Initial diagnosis as advanced stage	Progressed to advanced stage through intermediate stage	Progressed to advanced stage directly from early stage	*P*
**Number of patients**	123	56	38	29	
**Gender** [*n* (%)]					
Male	97 (79)	46 (82)	32 (84)	19 (66)	0.128
Female	26 (21)	10 (18)	6 (16)	10 (34)	
**Age, years** [*n* (%)]					
≤72	67 (54)	34 (61)	21 (55)	12 (41)	0.235
>72	56 (46)	22 (39)	17 (45)	17 (59)	
**HBV** [*n* (%)]					
Absent	100 (81)	42 (75)	32 (84)	26 (90)	0.223
Present	23 (19)	14 (25)	6 (16)	3 (10)	
**HCV** [*n* (%)]					
Absent	72 (59)	42 (75)	21 (55)	9 (31)	<0.001
Present	51 (41)	14 (25)	17 (45)	20 (69)	
**Alcohol abuse** [*n* (%)]					
Absent	108 (88)	48 (86)	33 (87)	27 (93)	0.600
Present	15 (12)	8 (14)	5 (13)	2 (7)	
**ECOG-PS >0** [*n* (%)]					
Absent	53 (43)	27 (48)	11 (29)	15 (52)	0.101
Present	70 (57)	29 (52)	27 (71)	14 (48)	
**Child–Pugh** [*n* (%)]					
A	87 (71)	32 (57)	29 (76)	26 (90)	0.005
B	36 (29)	24 (43)	9 (24)	3 (10)	
**Status of Intrahepatic lesions** [*n* (%)]					
None	13 (11)	3 (5)	1 (3)	9 (31)	<0.001
Without MVI	43 (35)	11 (20)	23 (61)	9 (31)	
With MVI	67 (54)	42 (75)	14 (37)	11 (38)	
**Maximum size of the intrahepatic lesion, >50 mm** [*n* (%)]					
Absent	74 (60)	19 (34)	29 (76)	26 (90)	<0.001
Present	49 (40)	37 (66)	9 (24)	3 (10)	
**Number of intrahepatic lesions, >7** [*n* (%)]					
Absent	59 (48)	23 (41)	15 (39)	21 (72)	0.011
Present	64 (52)	33 (59)	23 (61)	8 (28)	
**EHM** [*n* (%)]					
Absent	38 (31)	23 (41)	9 (24)	6 (21)	0.080
Present	85 (69)	33 (59)	29 (76)	23 (79)	
**AFP, ng/mL** [*n* (%)]					
≤400	61 (50)	24 (43)	20 (53)	17 (59)	0.350
>400	62 (50)	32 (57)	18 (47)	12 (41)	
**Pre-treatment** [*n* (%)]					
Absent	21 (17)	21 (38)	0 (0)	0 (0)	<0.001
Present	102 (83)	35 (63)	38 (100)	29 (100)	
**Initial dose of sorafenib, 800 mg/day** [*n* (%)]					
Absent	8 (7)	4 (7)	2 (5)	2 (7)	0.932
Present	115 (93)	52 (93)	36 (95)	27 (93)	
**Average daily dose** [*n* (%)]					
>400 mg	58 (47)	25 (45)	17 (45)	16 (55)	0.613
≤400 mg	65 (53)	31 (55)	21 (55)	13 (45)	

Abbreviations: HBV, hepatitis B virus; HCV, hepatitis C virus; ECOG-PS, Eastern Cooperative Oncology Group performance status; MVI, macrovascular invasion; EHM, extrahepatic metastasis; AFP, alpha-fetoprotein
