# Supplementary material for: Analysis of Sorafenib Outcome: Focusing on the Clinical Course in Patients with Hepatocellular Carcinoma
Source: PLoS One. 2016 Aug 18;11(8):e0161303. doi: 10.1371/journal.pone.0161303 (PMC4990272; doi:10.1371/journal.pone.0161303)
Supplement: S2 Table — (PDF) [file pone.0161303.s002.pdf]

S2 Table. Univariate analysis of survival in sorafenib-treated patients with advanced-stage hepatocellular carcinoma

Variables	Univariate analysis		*P*
	Hazard ratio	95% CI	
**Gender, male**			
Absent	Reference		
Present	0.907	0.540–1.522	0.711
**Age, >72 years**			
Absent	Reference		
Present	0.733	0.493–1.091	0.126
**HBV**			
Absent	Reference		
Present	1.313	0.784–2.201	0.301
**HCV**			
Absent	Reference		
Present	0.683	0.452–1.033	0.071
**Alcohol abuse**			
Absent	Reference		
Present	1.200	0.679–2.121	0.529
**ECOG-PS, >0**			
Absent	Reference		
Present	1.936	1.282–2.922	0.002
**Child–Pugh B**			
Absent	Reference		
Present	2.400	1.561–3.690	<0.001
**Status of intrahepatic lesions**			
None	0.610	0.282–1.320	0.210
Without MVI	Reference		
With MVI	2.200	1.408–3.437	0.001
**Maximum size of the intrahepatic lesion, >50 mm**			
Absent	Reference		
Present	1.528	1.021–2.288	0.039
**Number of intrahepatic lesions, >7**			
Absent	Reference		
Present	2.281	1.522–3.418	<0.001
**EHM**			
Absent	Reference		
Present	0.787	0.508–1.221	0.787
**AFP, >400 ng/mL**			
Absent	Reference		
Present	1.418	0.954–2.106	0.084
**Pre-treatment**			
Absent	Reference		
Present	0.673	0.391–1.157	0.152
**Stage progression process**			
Progress directly from early stage	Reference		
Others	1.762	1.060–2.858	0.022
**Average daily dose, ≤400 mg/day**			
Absent	Reference		
Present	0.935	0.629–1.388	0.737

Abbreviations: HBV, hepatitis B virus; HCV, hepatitis C virus; ECOG-PS, Eastern Cooperative Oncology Group performance status; MVI, macrovascular invasion; EHM, extrahepatic metastasis; AFP, alpha-fetoprotein
