# Supplementary material for: Analysis of Sorafenib Outcome: Focusing on the Clinical Course in Patients with Hepatocellular Carcinoma
Source: PLoS One. 2016 Aug 18;11(8):e0161303. doi: 10.1371/journal.pone.0161303 (PMC4990272; doi:10.1371/journal.pone.0161303)
Supplement: S3 Table — (PDF) [file pone.0161303.s003.pdf]

S3 Table. Baseline demographic data and patient characteristics at the time of starting sorafenib (intermediate-stage hepatocellular carcinoma patients)

	**All patients**	**Initial diagnosis as intermediate stage**	**Initial diagnosis as early stage**	***P***
**Number of patients**	67	32	35	
**Gender** [*n* (%)]				
Male	49 (73)	21(66)	28 (80)	0.270
Female	18 (27)	11 (34)	7 (20)	
**Age, years** [*n* (%)]				
≤72	36 (54)	16 (50)	20 (57)	0.628
>72	31 (46)	16 (50)	15 (43)	
**HBV** [*n* (%)]				
Absent	62 (93)	29 (91)	33 (94)	0.664
Present	5 (7)	3 (9)	2 (6)	
**HCV** [*n* (%)]				
Absent	23 (34)	17 (53)	6 (17)	0.004
Present	44 (66)	15 (47)	29 (83)	
**Alcohol abuse** [*n* (%)]				
Absent	65 (97)	31 (97)	34 (97)	1.000
Present	2 (3)	1 (3)	1 (3)	
**ECOG-PS >0** [*n* (%)]				
Absent	43 (64)	22 (69)	21 (60)	0.611
Present	24 (36)	10 (31)	14 (40)	
**Child–Pugh** [*n* (%)]				
A	51 (76)	25 (78)	26 (74)	0.780
B	16 (24)	7 (22)	9 (26)	
**Maximum size of the intrahepatic lesion, >50 mm** [*n* (%)]				
Absent	49 (73)	23 (72)	26 (74)	1.000
Present	18 (27)	9 (28)	9 (26)	
**Number of intrahepatic lesions, >7** [*n* (%)]				
Absent	18 (27)	9 (28)	9 (26)	1.000
Present	49 (73)	23 (72)	26 (74)	
**Sub-classification of BCLC B** [*n* (%)]				
B1	3 (4)	2 (6)	1 (3)	0.803
B2	29 (43)	15 (47)	14 (40)	
B3	11 (16)	5 (16)	6 (17)	
B4	24 (36)	10 (31)	14 (40)	
**AFP, ng/mL** [*n* (%)]				
≤400	45 (67)	22 (69)	23 (66)	1.000
>400	22 (33)	10 (31)	12 (34)	
**Pre-treatment** [*n* (%)]				
Absent	0 (0)	0 (0)	0 (0)	1.000
Present	67 (100)	32 (100)	35 (100)	
**Initial dose of sorafenib, 800 mg/day** [*n* (%)]				
Absent	5 (7)	1 (3)	4 (11)	1.000
Present	62 (93)	31 (97)	31 (89)	
**Average daily dose** [*n* (%)]				
>400 mg	40 (60)	20 (63)	20 (57)	0.804
≤400 mg	27 (40)	12 (37)	15 (43)	

Abbreviations: HBV, hepatitis B virus; HCV, hepatitis C virus; ECOG-PS, Eastern Cooperative Oncology Group performance status; MVI, macrovascular invasion; EHM, extrahepatic metastasis; BCLC, Barcelona clinic liver cancer; AFP, alpha-fetoprotein
