# Supplementary material for: Analysis of Sorafenib Outcome: Focusing on the Clinical Course in Patients with Hepatocellular Carcinoma
Source: PLoS One. 2016 Aug 18;11(8):e0161303. doi: 10.1371/journal.pone.0161303 (PMC4990272; doi:10.1371/journal.pone.0161303)
Supplement: S4 Table — (PDF) [file pone.0161303.s004.pdf]

S4 Table. Univariate analysis of survival in sorafenib-treated patients with intermediate-stage hepatocellular carcinoma

**Variables**	**Univariate analysis**		***P***
	**Hazard ratio**	**95% CI**	
**Gender, male**			
Absent	Reference		
Present	1.137	0.577–2.210	0.710
**Age, >72 years**			
Absent	Reference		
Present	0.818	0.456–1.468	0.501
**HBV**			
Absent	Reference		
Present	1.409	0.427–4.650	0.573
**HCV**			
Absent	Reference		
Present	0.960	0.521–1.768	0.895
**Alcohol abuse**			
Absent	Reference		
Present	0.458	0.062–3.363	0.443
**ECOG-PS, >0**			
Absent	Reference		
Present	1.810	1.009–3.245	0.046
**Child–Pugh B**			
Absent	Reference		
Present	2.083	1.092–3.974	0.026
**Maximum size of the intrahepatic lesion, >50 mm**			
Absent	Reference		
Present	1.302	0.684–2.479	0.421
**Number of intrahepatic lesions, >7**			
Absent	Reference		
Present	1.332	0.697–2.546	0.386
**AFP, >400 ng/mL**			
Absent	Reference		
Present	3.474	1.839–6.562	<0.001
**Stage progression process**			
Initial diagnosis as intermediate stage	Reference		
Initial diagnosis as early stage	0.958	0.536–1.711	0.884
**Average daily dose, ≤400 mg/day**			
Absent	Reference		
Present	1.223	0.666–2.247	0.517

Abbreviations: HBV, hepatitis B virus; HCV, hepatitis C virus; ECOG-PS, Eastern Cooperative Oncology Group performance status; MVI, macrovascular invasion; EHM, extrahepatic metastasis; AFP, alpha-fetoprotein
