# Supplementary material for: Analysis of Sorafenib Outcome: Focusing on the Clinical Course in Patients with Hepatocellular Carcinoma
Source: PLoS One. 2016 Aug 18;11(8):e0161303. doi: 10.1371/journal.pone.0161303 (PMC4990272; doi:10.1371/journal.pone.0161303)
Supplement: S5 Table — (PDF) [file pone.0161303.s005.pdf]

S5 Table. Progression pattern of sorafenib treatment, liver function deterioration at the end of sorafenib treatment, and post-sorafenib therapy

	N (%)	OS (months)	P (Log-rank)	Post-sorafenib therapy				
				BSC	Clinical trial	TACE	HAIC	Others
**Advanced stage**	123			79	15	23	5	1
**Intrahepatic lesion growth**								
Absent	65 (53)	5.9 (4.3–7.6)	0.239	49	4	10	1	1
Present	58 (47)	8.8 (4.5–13.1)		30	11	13	4	0
**Extrahepatic lesion growth**								
Absent	88 (72)	5.9 (3.5–8.2)	0.143	59	9	16	4	0
Present	35 (28)	15.3 (5.9–24.7)		20	6	7	1	1
**New intrahepatic lesion**								
Absent	79 (64)	8.0 (5.2–10.8)	0.613	57	10	7	4	1
Present	44 (36)	7.5 (4.4–10.6)		22	5	16	1	0
**New extrahepatic lesion**								
Absent	73 (59)	13.9 (7.5–20.2)	<0.001	40	13	16	3	1
Present	50 (41)	5.4 (3.4–7.5)		39	2	7	2	0
**Liver function deterioration**								
Absent	75 (61)	14.6 (9.1–20.2)	<0.001	34	14	21	5	1
Present	48 (39)	4.5 (3.4–5.7)		45	1	2	0	0
**Intermediate stage**	67			33	13	20	1	0
**Intrahepatic lesion growth**								
Absent	18 (27)	12.1 (6.8–17.5)	0.787	9	4	5	0	0
Present	49 (73)	13.8 (10.3–17.2)		24	9	15	1	0
**New intrahepatic lesion**								
Absent	27 (40)	23.2 (13.7–32.7)	0.017	10	7	10	0	0
Present	40 (60)	11.0 (8.3–13.8)		23	6	10	1	0
**New extrahepatic lesion**								
Absent	56 (84)	13.8 (11.2–16.3)	0.060	28	11	17	0	0
Present	11 (16)	7.1 (0.0–14.2)		5	2	3	1	0
**Liver function deterioration**								
Absent	54 (81)	14.5 (10.5–18.5)	<0.001	22	13	18	1	0
Present	13 (19)	7.1 (1.9–12.3)		11	0	2	0	0

Abbreviations: OS, overall survival; BSC, best supportive care; TACE, transcatheter arterial chemoembolization; HAIC, hepatic arterial infusion chemotherapy
