# Supplementary material for: Analysis of Sorafenib Outcome: Focusing on the Clinical Course in Patients with Hepatocellular Carcinoma
Source: PLoS One. 2016 Aug 18;11(8):e0161303. doi: 10.1371/journal.pone.0161303 (PMC4990272; doi:10.1371/journal.pone.0161303)
Supplement: S6 Table — (PDF) [file pone.0161303.s006.pdf]

**S6 Table**. Baseline demographic data and patient characteristics at the time of being diagnosed with intermediate-stage hepatocellular carcinoma (propensity score matched dataset)

| | Starting sorafenib before progressing to advanced stage | Starting sorafenib after progressing to advanced stage | *P* |
|---|---|---|---|
| **Number of patients** | 36 | 36 | |
| **Gender** [*n* (%)] | | | |
| Male | 31 (86) | 30 (83) | 1.000 |
| Female | 5 (14) | 6 (17) | |
| **Age, years** [*n* (%)] | | | |
| ≤72 | 22 (61) | 22 (61) | 1.000 |
| >72 | 14 (39) | 14 (39) | |
| **HBV** [*n* (%)] | | | |
| Absent | 35 (93) | 31 (86) | 0.199 |
| Present | 1 (7) | 5 (14) | |
| **HCV** [*n* (%)] | | | |
| Absent | 9 (25) | 20 (56) | 0.016 |
| Present | 27 (75) | 16 (44) | |
| **Alcohol abuse** [*n* (%)] | | | |
| Absent | 36 (100) | 31 (86) | 0.054 |
| Present | 0 (0) | 5 (14) | |
| **Child–Pugh** [*n* (%)] | | | |
| A | 35 (97) | 34 (94) | 1.000 |
| B | 1 (3) | 2 (6) | |
| **Maximum size of the intrahepatic lesion, >50 mm** [*n* (%)] | | | |
| Absent | 27 (75) | 26 (72) | 1.000 |
| Present | 9 (25) | 10 (28) | |
| **Number of intrahepatic lesions, >7** [*n* (%)] | | | |
| Absent | 25 (69) | 26 (72) | 1.000 |
| Present | 11 (31) | 10 (28) | |
| **Sub-classification of BCLC B** [*n* (%)] | | | |
| B1 | 13 (36) | 11 (31) | 0.539 |
| B2 | 23 (64) | 23 (64) | |
| B3 | 0 (0) | 1 (3) | |
| B4 | 0 (0) | 1 (3) | |
| **AFP, ng/mL** [*n* (%)] | | | |
| ≤400 | 24 (67) | 25 (69) | 1.000 |
| >400 | 12 (33) | 11 (31) | |
| **Initial diagnosis** [*n* (%)] | | | |
| Early stage | 18 (50) | 14 (39) | 0.477 |
| Intermediate stage | 18 (50) | 22 (61) | |
| **Initial dose of sorafenib, 800 mg/day** [*n* (%)] | | | |
| Absent | 1 (3) | 2 (6) | 1.000 |
| Present | 35 (97) | 34 (94) | |
| **Effectiveness of initial TACE in intermediate stage**[*n* (%)] | | | |
| Responder | 22 (61) | 24 (67) | 0.807 |
| Non-responder | 14 (39) | 12 (33) | |

Abbreviations: HBV, hepatitis B virus; HCV, hepatitis C virus; BCLC, Barcelona clinic liver cancer; AFP, alpha-fetoprotein; TACE, transcatheter arterial chemoembolization
