# Supplementary material for: Analysis of Sorafenib Outcome: Focusing on the Clinical Course in Patients with Hepatocellular Carcinoma
Source: PLoS One. 2016 Aug 18;11(8):e0161303. doi: 10.1371/journal.pone.0161303 (PMC4990272; doi:10.1371/journal.pone.0161303)
Supplement: S7 Table — (PDF) [file pone.0161303.s007.pdf]

S7 Table. Univariate analysis of survival after being diagnosed with intermediate stage hepatocellular carcinoma

Variables	Univariate analysis		***P***
	**Hazard ratio**	**95% CI**	
**Any patients (n = 105)**			
**Gender, male**			
Absent	Reference		
Present	0.880	0.507–1.527	0.650
**Age, >72 years**			
Absent	Reference		
Present	1.175	0.742–1.862	0.491
**HBV**			
Absent	Reference		
Present	0.980	0.471–2.040	0.958
**HCV**			
Absent	Reference		
Present	0.845	0.545–1.311	0.452
**Alcohol abuse**			
Absent	Reference		
Present	0.786	0.336–1.839	0.579
**Child–Pugh B**			
Absent	Reference		
Present	2.132	1.013–4.487	0.046
**Maximum size of the intrahepatic lesion, >50 mm**			
Absent	Reference		
Present	1.148	0.696–1.895	0.588
**Number of intrahepatic lesions, >7**			
Absent	Reference		
Present	1.376	0.881–2.150	0.160
**AFP, >400 ng/mL**			
Absent	Reference		
Present	1.501	0.927–2.431	0.099
**Initial diagnosis**			
Early stage	Reference		
Intermediate stage	1.146	0.741–1.773	0.541
**Effectiveness of initial TACE in intermediate stage**			
Responder	Reference		
Non-responder	2.959	1.845–4.745	< 0.001
**Administration timing of sorafenib**			
Conversion before progressing to advanced stage	Reference		
Conversion after progressing to advanced stage	1.692	1.083–2.641	0.021
**Propensity score matched dataset (n = 72)**			
**Gender, male**			
Absent	Reference		
Present	0.801	0.415–1.546	0.508
**Age, >72 years**			
Absent	Reference		
Present	1.127	0.663–1.918	0.658
**HBV**			
Absent	Reference		
Present	0.594	0.214–1.646	0.317
**HCV**			
Absent	Reference		
Present	0.948	0.560–1.603	0.841
**Alcohol abuse**			
Absent	Reference		
Present	0.987	0.356–2.738	0.981
**Child–Pugh B**			
Absent	Reference		
Present	2.504	0.772–8.122	0.126
**Maximum size of the intrahepatic lesion, >50 mm**			
Absent	Reference		
Present	1.406	0.768–2.574	0.269
**Number of intrahepatic lesions, >7**			
Absent	Reference		
Present	1.485	0.857–2.575	0.159
**AFP, >400 ng/mL**			
Absent	Reference		
Present	1.400	0.808–2.426	0.230
**Initial diagnosis**			
Early stage	Reference		
Intermediate stage	1.177	0.705–1.965	0.533
**Effectiveness of initial TACE in intermediate-stage**			
Responder	Reference		
Non-responder	2.584	1.475–4.526	0.001
**Administration timing of sorafenib**			
Conversion before progressing to advanced-stage	Reference		
Conversion after progressing to advanced-stage	1.765	1.051–2.962	0.032

Abbreviations: HBV, hepatitis B virus; HCV, hepatitis C virus; AFP, alpha-fetoprotein; TACE, transarterial chemoembolization.
